# Rapid Urine LAM Testing Improves Diagnosis of Expectorated Smear-Negative Pulmonary Tuberculosis in an HIV-endemic Region

**DOI:** 10.1038/srep19992

**Published:** 2016-02-11

**Authors:** Paul K. Drain, Lilishia Gounder, Faieza Sahid, Mahomed-Yunus S. Moosa

**Affiliations:** 1 Departments of Global Health, Medicine, and Epidemiology, University of Washington, Seattle, WA, USA; 2 Department of Surgery, Massachusetts General Hospital, Harvard Medical School, Boston, MA, USA; 3 Department of Virology, National Health Laboratory Service, Inkosi Albert Luthuli Central Hospital, Durban, South Africa; 4 Division of Infectious Diseases, Department of Internal Medicine, Chris Hani Baragwanath Academic Hospital, University of the Witwatersrand, Johannesburg, South Africa; 5 Department of Infectious Diseases, Nelson R. Mandela School of Medicine, University of KwaZulu-Natal, Durban, South Africa

## Abstract

We sought to determine if urine lipoarabinomannan (LAM) would improve diagnosis of pulmonary TB. We enrolled consecutive adults presenting with ≥2 TB-related symptoms, obtained one induced sputum sample for smear microscopy (AFB) and mycobacterial culture, and performed urine LAM testing (Determine^TM^ TB LAM, Alere). We used culture-confirmed pulmonary TB as the gold standard, and compared accuracy with area under receiver operating characteristic curves (AUROC). Among 90 participants, 82 of 88 tested (93%) were HIV-infected with a median CD4 168/mm^3^ (IQR 89–256/mm^3^). Diagnostic sensitivities of urine LAM and sputum AFB were 42.1% (95% CI 29.1–55.9%) and 21.1% (95% CI 11.4–33.9%), and increased to 52.6% (95% CI 39.0–66.0%) when combined. Sensitivity of LAM increased significantly among participants with a lower Karnofsky Performance score, anemia, hypoalbuminemia, and higher C-reactive protein. Combining LAM with AFB had an AUROC = 0.68 (95% CI 0.59–0.77), significantly better than AFB alone (AUROC=0.58; 95% CI 0.51–0.64). The combination of LAM and AFB was significantly better than AFB alone among patients with Karnofsky Performance score ≤90, hemoglobin ≤10 g/dL, albumin ≤25 g/L, C-reactive protein ≥25 mg/L, or CD4 <200/mm^3^. Urine LAM testing may be most beneficial among patients with functional impairment, elevated inflammatory markers, or greater immunosuppression.

Over 9 million people develop active tuberculosis (TB) disease each year, and TB is responsible for more deaths each year than any other infectious disease^1^. In addition, approximately one-third of cases are either not diagnosed or not recorded by national TB programs^1^,^2^. Performance of existing tests for diagnosing active TB have been suboptimal in HIV-endemic regions^3^,^4^. Sputum smear microscopy for acid-fast bacilli (AFB) has poor diagnostic sensitivity^5^,^6^,^7^, and mycobacterial sputum culture has limited availability in resource-limited settings^8^,^9^. The Xpert MTB/RIF assay has improved the diagnosis of active pulmonary TB, but the assay’s cost and reliance on electricity make the test less practical for use at the clinical point-of-care^10^,^11^. Thus, developing better diagnostic algorithms that utilizes existing, conventional tests or novel point-of-care assays for improved detection of pulmonary TB remains a global public health priority^1^,^2^.

Lipoarabinomannan (LAM) is a lipopolysaccharide in the cell wall of *M. tuberculosis* that is released from organisms and excreted in urine^12^. A laboratory-based urine LAM-ELISA test had good diagnostic specificity, but poor sensitivity for diagnosing active TB disease^13^,^14^,^15^. A rapid, disposable lateral flow urine LAM assay can be conducted at the clinical point-of-care in 25 minutes^16^ and be used as a biomarker to predict mortality outcomes during anti-TB therapy^17^,^18^. Several studies, including our own, have demonstrated that the rapid urine LAM assay has adequate diagnostic specificity, but poor sensitivity^16^,^19^,^20^,^21^,^22^,^23^,^24^, which severely limits its utility as a solitary diagnostic test.

Since point-of-care diagnostics have many appealing properties for use in resource-limited settings^25^, we sought to determine if rapid urine LAM and induced sputum smear microscopy could improve diagnostic accuracy among adults with presumed sputum smear-negative pulmonary TB. Furthermore, since many point-of-care diagnostic tests, including C-reactive protein (CRP), hemoglobin, and CD4 count, have now emerged^25^, we additionally wanted to assess whether a combination of clinical, inflammatory, and immunologic markers would improve the diagnostic value of rapid urine LAM testing.

## Methods

### Study design and participant selection

We conducted a prospective cohort study of adults who were smear-negative by expectorated sputum, but presumed to have pulmonary TB on the basis of symptoms, at King Edward VIII Hospital in Durban, South Africa from November 2008 to December 2009^17^. We enrolled consecutive participants ≥18-years-old with AFB-smear negative on expectorated sputum and at least two of four TB-related symptoms (cough, fever, weight loss, night sweats) for at least two weeks. Participants were determined to have smear-negative disease if 2 expectorated sputa samples, one of which was performed by our research lab, were confirmed AFB-smear negative. The time between eligibility and study entry was less than 7 days for all participants. Since many participants were unaware of their HIV status at clinical presentation, we offered HIV testing but did not include HIV-infected as either an inclusion or exclusion criterion. We excluded patients who had a Karnofsky Performance score <50 in order to target a relatively well, ambulant population where the majority of smear-negative disease is seen, had taken anti-TB medications within the prior three months, or had respiratory distress, chronic obstructive pulmonary disease, or cardiac failure. The Biomedical Research Ethics Committee of the University of KwaZulu-Natal approved the study, the research methods were carried out in accordance with the approved guidelines, and all participants provided written informed consent.

### Study procedures

Upon enrollment, we obtained a detailed social and medical history, assessed Karnofsky Performance score. Although patients did not require hospitalization, we admitted participants for overnight observation in order to obtain one early-morning sputum sample by induction with an ultrasonic nebulizer using 5% hypertonic saline and obtained a urine sample. Respiratory samples were tested by smear microscopy for AFB and cultured for *Mycobacterium tuberculosis*. Urine samples were obtained at the time of enrollment in a sterile container for LAM testing. Participants had chest radiography and additional laboratory testing, including hemoglobin, serum albumin, and CRP. Participants were offered tuberculin skin testing with purified protein derivative and HIV screening, and CD4 count testing if HIV-infected. Since participants were presumed to have had active pulmonary TB, all participants were started on empiric therapy after collection of respiratory, blood and urine specimens. Participants were referred for initiation of antiretroviral therapy if HIV-infected, in accordance with South African guidelines^26^.

### Tuberculosis Testing

Certified technologists performed sputum smear microscopy for AFB and mycobacterial culture from respiratory samples in a reference laboratory at the University of KwaZulu-Natal. Sputum samples were decontaminated with N-acetyl L Cysteine and NaOH to a final concentration of 1.25% before being centrifuged at 3,000 rpm for 20 minutes and resuspended in one milliliter of 7H9 broth. Smear microscopy was performed using both Ziehl-Neelson and Auramine stains, and considered positive if either stain revealed AFB. Mycobacterial culture was performed by inoculating Middlebrook 7H11 solid agar medium, and inoculating 0.5 milliliter in liquid mycobacterial growth indicator tubes (MGIT™) in an automated Bactec™ 960 instrument (BD; Franklin Lakes, USA). Culture plates were read at 3 and 6 weeks, and *M. tuberculosis* was identified from solid or liquid cultures using niacin and nitrate testing. The study was conducted and specimens were collected before the Xpert MTB/RIF assay became available in South Africa.

Urine LAM assay testing was performed on urine samples stored in a −20 **°**C frost-free freezer using the Determine™ TB LAM test (Alere Inc., Waltham, USA) in accordance with the manufacturer’s specifications. Urine LAM assay kits from Manufacturer Lot Numbers 110512, 120215, and 120222 were maintained at room temperature (15–26 °C). We brought urine specimens to room temperature, centrifuged samples at 10,000 rpm for five minutes, and used a sterile pipette to transfer 60 microliters of the clear supernatant to the test strip. Two readers (PKD and LG) interpreted the test results after 25 minutes and reached consensus if there was disagreement. LAM assays without positive control lines were considered ‘invalid’ and repeated, but we did not observe any test strip failures. Positive LAM assays were graded from low band intensity (1+ grade) to high band intensity (5+ grade), according to the manufacturer’s original reference card with five positive categories. We included the 1+ grade, since the data were collected before the manufacturer modified the reference card. Operators and readers were blinded to participant information during urine LAM and TB testing.

### Statistical Analysis

We used sputum culture for *M. tuberculosis* as the gold-standard diagnostic test for active pulmonary TB, and followed STARD guidelines for reporting diagnostic accuracy^27^. We used Fisher’s Exact test for categorical variables and t-test for continuous measures to compare characteristics between TB culture-negative and –positive participants. We calculated sensitivity, specificity, predictive values (positive and negative) for sputum AFB and urine LAM assays, alone and in combination, for diagnosing sputum culture-confirmed pulmonary TB. We evaluated the performance of urine LAM in participants categorized according to clinical manifestations, levels of inflammatory markers, and immunological status. We used area under receiver operating characteristic curves (AUROC) to evaluate overall diagnostic accuracy of sputum smear and urine LAM, individually or in combination. Since other rapid, point-of-care tests, such as hemoglobin, CRP, and CD4 count, might be used in this setting, we incorporated these in AUROC analyses. We compared AUROC estimates using the DeLong test^28^, reported two-tailed p-values using α = 0.05, and conducted analyses using SAS 9.4 (Cary, NC, USA).

## Results

Among 90 participants enrolled, mean age was 36.9 years (standard deviation ±9.3 years) and 44 participants (49.1%) were female (Table 1). Fifty-seven participants (63.3%) were diagnosed with culture-confirmed pulmonary TB. Of 85 participants tested, forty-five (52.9%) were tuberculin skin test positive. Among 88 participants consented and tested for HIV, 82 (93.2%) were HIV-infected with a median CD4 cell count of 168/mm^3^ (interquartile range 89–256/mm^3^). Culture-positive participants had significantly lower Karnofsky Performance scores, hemoglobin, and serum albumin, but a significantly higher number of TB-related symptoms and CRP concentrations. There were no significant differences in tuberculin skin test positivity, HIV infection, or CD4 count between TB culture-negative and -positive participants.

### Sputum Smear and Urine LAM Testing

Induced sputum samples from 14 participants (15.6%) were AFB smear-positive. The overall sensitivity and specificity of one sputum AFB smear for diagnosing culture-confirmed pulmonary TB was 21.1% (95% CI 11.4–33.9%) and 93.9% (95% CI 79.8–99.3%) (Table 2). The urine LAM assay had a sensitivity and specificity of 42.1% (95% CI 29.1–55.9%) and 84.9% (95% CI 68.1–94.9%). Using a LAM grade ≥2+ threshold decreased sensitivity to 22.8% (95% CI 12.7–35.8%), and improved specificity to 97.0% (95% CI 84.2–99.9%). Overall, using higher band intensities for a positive LAM test threshold reduced diagnostic sensitivity, but improved specificity.

There was poor agreement between the urine LAM assay and sputum AFB (κ = 0.08; p = 0.28). Eight participants were sputum AFB positive and urine LAM negative, while 23 participants were urine LAM positive and sputum AFB negative. When using a combined diagnostic strategy of a positive sputum AFB smear or a positive urine LAM test, diagnostic sensitivity increased to 52.6% (95% CI 39.0–66.0%), but specificity decreased to 78.8% (95% CI 61.1–91.0%). When using a higher threshold for a positive LAM (grade ≥2+), sensitivity decreased to 38.6% (95% CI 26.0–52.4%), while specificity was 90.9% (95% CI 75.7–98.1%).

### Urine LAM Assay by Clinical, Inflammatory, and Immunologic Characteristics

The diagnostic sensitivity of the urine LAM assay was significantly different when applied to subgroups with particular clinical and/or laboratory characteristics (Table 3). The urine LAM assay had higher diagnostic sensitivity among participants with a lower Karnofsky Performance score, lower serum hemoglobin, and higher CRP, while there were no significant differences by number of TB-related symptoms. Diagnostic sensitivity of urine LAM with 1+ grade among participants with a CD4 count less than the median (168/mm^3^) was 58.6% (95% CI 38.9–76.5%), which was significantly better than among those with a CD4 above the median (25.0%; 95% CI 9.8–46.7%). Sensitivity of urine LAM was also significantly higher among participants with a serum albumin <26 g/L (67.9%; 95% CI 47.7–84.1%), when compared to those with a serum albumin ≥26 g/L (17.2%; 95% CI 5.6–35.8%). The results were similar using a higher intensity threshold for the urine LAM assay (grade ≥2+).

There were also significant differences in clinical and laboratory parameters when comparing positive low-grade (1+) and high-grade (≥2+) LAM assay results (Table 4). Participants with a high-grade positive urine LAM result had significantly lower hemoglobin and serum albumin levels, compared to those with a low-grade positive result. Participants with low-grade positive urine LAM were also more likely to be tuberculin skin test positive (p = 0.02). There were no significant differences between those with low- and high-grade positive LAM tests in sputum AFB smear positivity and mean CD4 count.

### Value of Using a Combination of Sputum Smear and Urine LAM Testing

**Figure 1:**
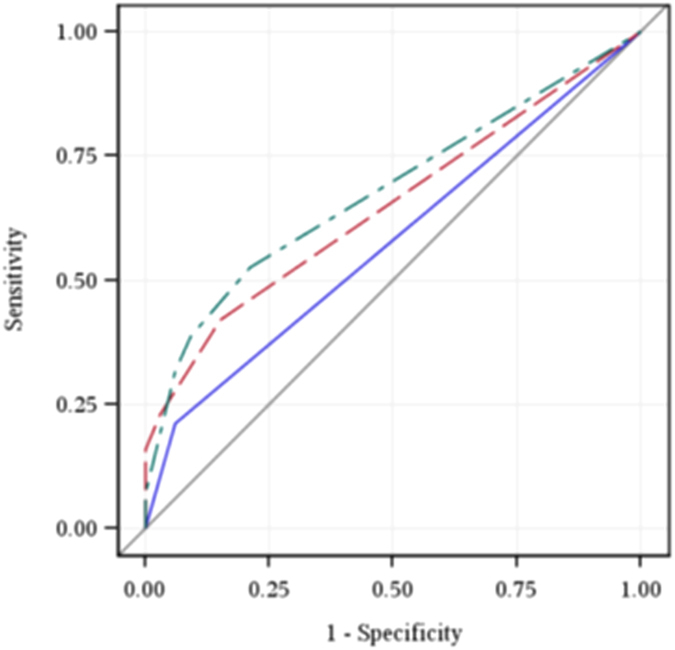
ROC curves for induced sputum AFB smear (solid blue line), urine LAM assay (dashed red line), and combination of induced sputum AFB smear or urine LAM assay (irregular green line) for diagnosing culture-confirmed pulmonary TB among the entire cohort.

**Figure 2:**
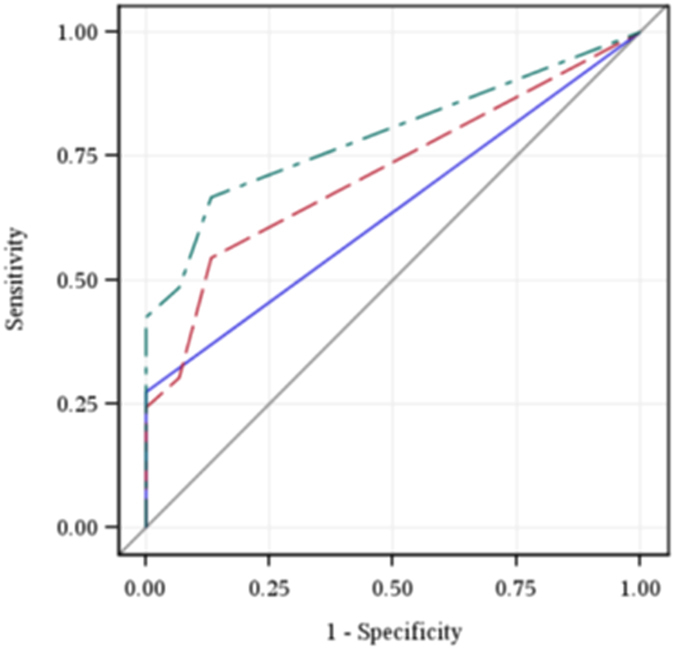
ROC curves for induced sputum AFB smear (solid blue line), urine LAM assay (dashed red line), and combination of induced sputum AFB smear or urine LAM assay (irregular green line) for diagnosing culture-confirmed pulmonary TB among those with a CD4 count <200/mm^3^.

We assessed overall diagnostic accuracy of various testing strategies for sputum AFB and urine LAM testing in our cohort (Table 5). Induced sputum AFB smear alone had an AUROC of 0.58 (95% CI 0.51–0.64) and the urine LAM assay alone had an AUROC of 0.65 (95% CI 0.56–0.73). Combining urine LAM with sputum AFB had an AUROC = 0.68 (95% CI 0.59–0.77), which was significantly better than induced sputum AFB smear alone (p = 0.006) (Fig. 1). Stratifying results by various clinical and laboratory characteristics improved diagnostic outcomes for urine LAM testing, but not for sputum smear testing. The diagnostic value of urine LAM in patients categorized by severity of clinical illness, levels of inflammatory markers and degree of immune suppression is also demonstrated by AUROC analysis (Table 5). The highest AUROC values were for performing urine LAM alone for participants with albumin ≤25 g/L (AUROC = 0.79; 95% CI 0.66–0.92), and both urine LAM and smear AFB for those with a CD4 <200/mm^3^ (AUROC = 0.79; 95% CI 0.69–0.90) (Fig. 2). The combination of urine LAM and sputum AFB was significantly better than sputum AFB testing alone across all categories of patients with functional impairment, elevated inflammation, and greater immunosuppression.

## Discussion

In this study of adults presenting with expectorated smear-negative sputum and symptomatic TB disease, the urine LAM assay was beneficial in conjunction with induced sputum smear microscopy for diagnosing culture-confirmed pulmonary TB. Overall, a darker band intensity of the urine LAM assay had improved specificity, or lower false positive rate, but with reduced diagnostic sensitivity. Rapid urine LAM testing was more sensitive at diagnosing TB in patients with more advanced disease, as suggested by presence of severe anemia, lower albumin levels, higher acute phase reactants, and a poorer functional state. Conducting the urine LAM assay in combination with sputum AFB smear improved diagnostic accuracy, and was most valuable among patients with functional impairment (low Karnofsky Performance score), hypoalbuminemia, or a low CD4 count (<200/mm^3^).

The diagnostic accuracy of the urine LAM assay has been reported in hospital-based settings, but not from an ambulatory population with expectorated smear-negative pulmonary TB. Studies of HIV-infected patients presenting to a clinic or hospital with TB-related symptoms have reported a diagnostic sensitivity of urine LAM between 62–69%^16^,^19^,^20^,^21^,^22^,^23^,^24^, which was higher than our study. This difference may be due to these studies including participants with smear-positive by expectorated sputum, which might be a marker for a high bacillary load, as well as sicker patients. Since our data showed better overall diagnostic accuracy for combined urine LAM testing among sicker patients, the LAM test might have greater value in diagnosing TB in patients that require hospitalization for advanced TB. Other studies have also shown the urine LAM assay to have improved sensitivity among immunosuppressed adults^16^,^23^,^24^, and among patients with elevated inflammation^29^. While our study was representative of patients presenting with TB-related symptoms regardless of HIV status, urine LAM has limited diagnostic value among HIV-negative people^30^. Our study builds upon previous studies by demonstrating that urine LAM adds to the overall diagnostic accuracy among adults who present to the hospital with TB-related symptoms, but have an initial negative expectorated sputum AFB smear microscopy.

The urine LAM assay is an imperfect pathogen biomarker for an active TB infection, but may provide additional diagnostic value in certain clinical settings. The increased specificity with darker LAM band intensity suggests a greater bacillary load of TB, which is consistent with our previous finding that LAM decreases with anti-TB therapy^17^. The finding that people with a higher LAM grade had significantly lower hemoglobin and albumin levels, but higher CRP levels, further suggests that a darker LAM band is associated with a greater overall burden of TB disease. Since the LAM band intensity ≥3+ had a diagnostic specificity of 100%, or no false positive results, the presence of a strong LAM band intensity provides good diagnostic value to clinicians. However, with a peak sensitivity of only 68% among the most severely ill patients, the rapid urine LAM test should not be used to rule out or exclude active TB disease. Further refinement of the existing LAM assay, as well as algorithms incorporating the urine LAM test with other rapid diagnostic TB tests, may be valuable. In addition, the positive predictive value of urine LAM testing will depend upon the prevalence of TB disease in the population, or the pre-test probability of disease, which will be different for an ambulatory clinical population as well as various geographical regions.

Among the entire cohort, the urine LAM assay improved the overall diagnostic accuracy of induced sputum AFB testing alone. Since the benefit was most pronounced among patients with functional impairment, more systemic inflammation, and a greater immunodeficiency, hospital-based settings with existing sputum AFB capacity should consider adding rapid urine LAM testing. The overall diagnostic accuracy of urine LAM was superior to AFB smear of induced sputa, suggesting that implementation of point-of-care urine LAM testing would be valuable. However, similar to smear microscopy, clinicians should not rely upon negative urine LAM testing alone to exclude active TB disease in HIV-endemic regions.

Since sputum culture is an imperfect gold standard test for pulmonary TB and we did not perform a complete evaluation for extrapulmonary disease, the specificity of urine LAM should be interpreted with caution. A more comprehensive assessment for active extrapulmonary TB infection may reduce the misclassification of positive urine LAM results among people with culture-negative TB disease. However, urine LAM testing identified significantly more people with active TB, has a lower occupational risk to health workers, and may be easier to implement than sputum AFB microscopy. In addition, sputum induction is not generally practiced in resource-limited TB-endemic regions, including South Africa, due to a lack of equipment and trained respiratory therapists. Since a challenge in the TB-endemic has been identifying TB-infected people, perhaps a small percentage of calculated overtreatment may be acceptable under better performing rapid TB diagnostic strategies.

Our study has several strengths and limitations. Primary strengths of this study were recruiting sequential patients with symptoms highly suggestive of active pulmonary TB, but with expectorated sputum that was AFB smear negative, comparing diagnostic strategies using several clinical parameters and inflammatory biomarkers, and assessing both liquid and solid mycobacterial culture as the gold standard. Limitations included obtaining a single respiratory sputum sample and not performing comprehensive measures for extrapulmonary TB, including mycobacterial blood cultures, both of which may have missed patients with active TB. Urine LAM testing was performed on thawed, not fresh, urine samples, and it is unclear if this could impact urine LAM results. We also did not evaluate sputum gram stain or epithelial cell count to determine the adequacy or contamination of each sputum specimen. However, we did perform nebulized sputum induction, which has been reported to being equivalent to lower respiratory tract sampling by bronchoalveolar lavage^31^. We evaluated diagnostic tests among patients presumed to have TB in a region with a high prevalence of TB and HIV, which limits the study’s generalizability.

In conclusion, the urine LAM assay is a relatively inexpensive test (US $2.66/test on 7 January 2015)^32^ that can be readily performed to identify more cases of HIV-associated pulmonary TB^33^. An overnight hospital stay is not required to obtain an early morning induced sputum, and the value of testing might be optimized with both early morning sputum and urine LAM testing. Urine LAM testing had most benefit among patients with functional impairment, elevated inflammation, or greater immunosuppression, who likely had a higher TB bacillary load, and may be best utilized in conjunction with sputum AFB smear for patients presenting with TB-related symptoms in HIV-endemic settings. Additional studies are needed to model these diagnostic parameters into an optimal algorithm with various levels of disease prevalence.

## Additional Information

**How to cite this article**: Drain, P. K. *et al*. Rapid Urine LAM Testing Improves Diagnosis of Expectorated Smear-NegativePulmonary Tuberculosis in an HIV-endemic Region. *Sci. Rep.* **6**, 19992; doi: 10.1038/srep19992 (2016).

## Figures and Tables

**Table 1:** Baseline cohort characterstics by sputum culture for pulmonary tuberuclosis.

	Culture-Negative Mean ± SD or N (%) (N = 33)	Culture-Positive Mean ± SD or N (%) (N = 57)	p-value
Demographics			
Age (years)	36.4 ± 7.6	37.1 ± 10.3	0.71
Female gender	18 (54.6)	28 (49.1)	0.66
Clinical			
Weight (kilograms)	59.0 ± 7.7	59.4 ± 11.9	0.87
BMI (kilograms/meter^2^)	21.7 ± 2.6	21.8 ± 3.9	0.88
Karnofsky Performance score			0.006
100	26 (81.3)	27 (48.2)	
90	3 (9.4)	16 (28.6)	
≤80	3 (9.4)	13 (23.2)	
Number of TB-related symptoms*			0.01
4 symptoms	25 (75.8)	50 (87.7)	
3 symptoms	3 (9.1)	7 (12.3)	
2 symptoms	5 (15.2)	0 (0)	
Laboratory Testing			
Hemoglobin (g/dL)	10.8 ± 2.2	9.3 ± 2.1	0.003
Albumin (g/L)	29.3 ± 6.0	24.7 ± 4.3	0.0005
C-reactive protein (mg/L)	41.8 ± 49.9	77.3 ± 66.4	0.006
Tuberculosis Testing (n = 85)			
Tuberculin skin test positive	16 (48.5)	29 (55.8)	0.66
HIV Testing (n = 88)			
HIV-infected	29 (87.9)	53 (96.4)	0.19
CD4 count/mm^3^ among HIV+	205 ± 141	195 ± 177	0.78

BMI = body mass index; SD = standard deviation; TB = tuberculosis.

*Includes cough, fever, night sweats, weight loss.

**Table 2:** Diagnostic accuracy for induced sputum AFB smear and urine LAM assay for diagnosing culture-confirmed pulmonary TB.

	Sensitivity		Specificity		Positive PV	Negative PV
	N/N	% (95% CI)	N/N	% (95% CI)	% (95% CI)	% (95% CI)
Sputum AFB Smear	12/57	21.1 (11.4–33.9)	31/33	93.9 (79.8–99.3)	85.7 (57.2–98.2)	40.8 (29.7–52.7)
Urine LAM Assay						
≥1+ grade	24/57	42.1 (29.1–55.9)	28/33	84.9 (68.1–94.9)	82.8 (64.2–94.2)	45.9 (33.1–59.2)
≥2+ grade	13/57	22.8 (12.7–35.8)	32/33	97.0 (84.2–99.9)	92.9 (66.1–99.8)	42.1 (30.9–54.0)
≥3+ grade	9/57	15.8 (7.5–27.9)	33/33	100 (89.4–100)	100 (66.4–100)	40.7 (30.0–52.2)
≥4+ grade	7/57	12.3 (5.1–23.7)	33/33	100 (89.4–100)	100 (59.0–100)	39.8 (29.2–51.1)
5+ grade	4/57	7.0 (2.0–17.0)	33/33	100 (89.4–100)	100 (39.8–100)	38.4 (28.1–49.5)
Sputum AFB or Urine LAM ≥1+ grade	30/57	52.6 (39.0–66.0)	26/33	78.8 (61.1–91.0)	81.1 (64.8–92.0)	49.1 (35.1–63.2)
Sputum AFB or Urine LAM ≥2+ grade	22/57	38.6 (26.0–52.4)	30/33	90.9 (75.7–98.1)	88.0 (68.8–97.5)	46.2 (33.7–59.0)

AFB = acid-fast bacilli; CI = confidence interval; LAM = lipoarabinomannan; PV = predictive value; TB = tuberculosis.

**Table 3:** Diagnostic accuracy of urine LAM for diagnosing culture-confirmed pulmonary TB by clinical and laboratory results*.

	Sensitivity		
	N/N	% (95% CI)	p-value
Urine LAM grade ≥1+			
Karnofsky Performance score ≤90	18/30	60.0 (40.6–77.3)	0.007
Karnofsky Performance score 100	6/27	22.2 (8.6–42.3)	
Hemoglobin <9.8 g/dL	20/34	58.8 (40.7–75.4)	0.003
Hemoglobin ≥9.8 g/dL	4/23	17.4 (4.6–38.8)	
Albumin <26 g/L	19/28	67.9 (47.7–84.1)	0.0001
Albumin ≥26 g/L	5/29	17.2 (5.6–35.8)	
C-reactive protein <46 mg/L	5/22	22.7 (7.8–45.4)	0.03
C-reactive protein ≥46 mg/L	18/34	52.9 (35.1–70.2)	
CD4 <168/mm^3^	17/29	58.6 (38.9–76.5)	0.03
CD4 ≥168/mm^3^	6/24	25.0 (9.8–46.7)	
Urine LAM grade ≥2+			
Karnofsky Performance score ≤90	12/30	40.0 (22.7–59.4)	0.001
Karnofsky Performance score 100	1/27	3.7 (0.1–19.0)	
Hemoglobin <9.8 g/dL	13/34	38.2 (22.2–56.4)	0.0007
Hemoglobin ≥9.8 g/dL	0/23	0	
Albumin <26 g/L	13/28	46.4 (27.5–66.1)	0.0001
Albumin ≥26 g/L	0/29	0	
C-reactive protein <46 mg/L	1/22	4.6 (0.1–22.8)	0.02
C-reactive protein ≥46 mg/L	11/34	32.4 (17.4–50.5)	
CD4 <168/mm^3^	10/29	34.5 (17.9–54.3)	0.11
CD4 ≥168/mm^3^	3/24	12.5 (2.7–32.4)	

CI = confidence interval; LAM = lipoarabinomannan; TB = tuberculosis.

*The cut-offs for each parameter was based on the median value.

**Table 4:** Clinical and laboratory test results by positive urine LAM grade.

	Urine LAM ≥1+ grade Mean ± SD or N (%) (N = 15)	Urine LAM ≥2+ grade Mean ± SD or N (%) (N = 14)	p-value
Clinical			
Weight (kilograms)	58.0 ± 6.1	59.2 ± 15.6	0.80
BMI (kilograms/meter^2^)	20.9 ± 2.8	22.1 ± 4.5	0.43
Karnofsky Performance score			0.12
100	8 (53.3)	2 (15.4)	
90	3 (20.0)	3 (23.1)	
≤80	4 (26.7)	8 (61.5)	
Number of TB-related symptoms*			1.0
4 symptoms	14 (93.3)	13 (92.9)	
3 symptoms	1 (6.7)	1 (7.1)	
2 symptoms	0 (0)	0 (0)	
Laboratory Testing			
Hemoglobin (g/dL)	9.1 ± 1.9	7.4 ± 1.4	0.007
Albumin (g/L)	25.1 ± 3.3	20.5 ± 2.3	0.0002
C-reactive protein (mg/L)	67.5 ± 41.7	119.1 ± 88.0	0.07
Tuberculosis Testing			
Tuberculin skin test positive (n = 85)	10 (66.7)	2 (16.7)	0.02
Sputum AFB smear positive	3 (20.0)	3 (21.4)	1.0
HIV Testing (n=88)			
CD4 cells/mm^3^ among HIV+	152 ± 107	143 ± 154	0.85

AFB = acid-fast bacilli; BMI = body mass index; SD = standard deviation; TB = tuberculosis.

*Includes cough, fever, night sweats, weight loss.

**Table 5:** Comparison of area under receiver operating characteristic curves (AUROC) to assess overall diagnostic accuracy of testing strategies for diagnosing culture-confirmed pulmonary TB.

	AUROC (95% CI)	p-value vs. Sputum AFB smear
TB Testing Strategies (n = 90)		
Sputum AFB smear positive	0.58 (0.51–0.64)	—
Urine LAM assay positive	0.65 (0.56–0.73)	0.189
Sputum AFB or LAM assay positive	0.68 (0.59–0.77)	0.006
If Karnofsky Performance score ≤90 (n = 37)		
Sputum AFB smear positive	0.58 (0.52–0.65)	—
Urine LAM assay positive	0.76 (0.63–0.89)	0.013
Sputum AFB or LAM assay positive	0.78 (0.66–0.90)	0.002
If Hemoglobin ≤10 g/dL (n = 49)		
Sputum AFB smear positive	0.58 (0.47–0.69)	—
Urine LAM assay positive	0.69 (0.55–0.82)	0.227
Sputum AFB or LAM assay positive	0.71 (0.56–0.85)	0.026
If Albumin ≤25 g/L (n = 37)		
Sputum AFB smear positive	0.57 (0.43–0.71)	—
Urine LAM assay positive	0.79 (0.66–0.92)	0.027
Sputum AFB or LAM assay positive	0.77 (0.61–0.94)	<0.001
If C-reactive protein ≥25 mg/L (n = 60)		
Sputum AFB smear positive	0.59 (0.50–0.68)	—
Urine LAM assay positive	0.66 (0.54–0.78)	0.401
Sputum AFB or LAM assay positive	0.71 (0.58–0.83)	0.028
If CD4 <200/mm^3^ (n = 48)		
Sputum AFB smear positive	0.64 (0.56–0.71)	--
Urine LAM assay positive	0.72 (0.60–0.83)	0.265
Sputum AFB or LAM assay positive	0.79 (0.69–0.90)	0.003

AFB = acid-fast bacilli; AUROC = area under receiver operating characteristic curve; CI = confidence interval; LAM = lipoarabinomannan; TB = tuberculosis.
